# Persistence of Pathogens on Inanimate Surfaces: A Narrative Review

**DOI:** 10.3390/microorganisms9020343

**Published:** 2021-02-09

**Authors:** Jan Erik Wißmann, Lisa Kirchhoff, Yannick Brüggemann, Daniel Todt, Joerg Steinmann, Eike Steinmann

**Affiliations:** 1 Department for Molecular and Medical Virology, Medical Faculty, Ruhr University Bochum, 44801 Bochum, Germany; Jan.wissmann@ruhr-uni-bochum.de (J.E.W.); Yannick.Brueggemann@ruhr-uni-bochum.de (Y.B.); Daniel.todt@ruhr-uni-bochum.de (D.T.); 2 Institute of Medical Microbiology, University Hospital Essen, University of Duisburg-Essen, 45147 Essen, Germany; Lisa.Kirchhoff@uk-essen.de; 3 European Virus Bioinformatics Center (EVBC), 07743 Jena, Germany; 4 Institute of Clinical Hygiene, Medical Microbiology and Infectiology, Klinikum Nürnberg, Paracelsus Medical University, 90471 Nürnberg, Germany

**Keywords:** viruses, bacteria, fungi, inanimate surfaces, stability

## Abstract

For the prevention of infectious diseases, knowledge about transmission routes is essential. In addition to respiratory, fecal–oral, and sexual transmission, the transfer of pathogens via surfaces plays a vital role for human pathogenic infections—especially nosocomial pathogens. Therefore, information about the survival of pathogens on surfaces can have direct implications on clinical measures, including hygiene guidelines and disinfection strategies. In this review, we reviewed the existing literature regarding viral, bacterial, and fungal persistence on inanimate surfaces. In particular, the current knowledge of the survival time and conditions of clinically relevant pathogens is summarized. While many pathogens persist only for hours, common nosocomial pathogens can survive for days to weeks under laboratory conditions and thereby potentially form a continuous source of transmission if no adequate inactivation procedures are performed.

## 1. Introduction

Many pathogens require a living host to survive, while others may be able to persist in a dormant state outside of a living host. Nonetheless, all pathogens require mechanisms to transfer from one host to another. Understanding how infectious pathogens spread is critical to prevent the transmission of infectious diseases. In addition to respiratory, fecal-oral and sexual transmission, the transmission of pathogens between hosts via fomites plays an important role in the spread of infectious diseases. Surface contamination has been described before [1,2,3] and has also been demonstrated via experimental modelling [4,5,6]. Importantly, the origin of various outbreaks of infectious diseases has been linked to fomite-mediated transmission [1,7,8,9,10,11,12,13,14,15,16,17]. Given that humans spend most of their time indoors [16] and are therefore in constant contact with potentially contaminated surfaces, detailed knowledge about the survival of pathogens on fomites plays a vital role in the understanding—and hence control—of infectious diseases. Hygiene measures based on this knowledge are therefore an essential part of infection prevention and are important in lowering the risk of pathogen transmission. In particular, in hospitals and other healthcare facilities, control of infections is mandatory to prevent nosocomial outbreaks. Consequently, the persistence of different nosocomial pathogens on inanimate surfaces has been extensively studied. However, the available experimental data on the survival of different pathogens has not been summarized in recent years. The aim of this review is to provide a comprehensive overview of the current scientific literature and evidence regarding the persistence of different nosocomial pathogens on inanimate surfaces.

## 2. Methods

We performed literature research as follows: Scientific papers on the basis of experimental evidence were searched on MedLine using a combination of the following search terms: ((persistence OR survival) AND (surface OR fomite) AND (bacteria OR virus OR pathogen)), ((persistence OR survival) AND (surface OR fomite) AND (bacteria OR virus OR pathogen) AND transmission)), ((persistence OR survival) AND (surface OR fomite) AND (bacteria OR virus OR pathogen) AND nosocomial)). Additionally, as filter we used “Species: humans”. The authors were not blinded and selected the used publications manually. Literature research was done between January and July 2020. Furthermore, we selected publications that appeared in citations. Only human pathogens were chosen and clustered into DNA or RNA viruses; gram-positive, gram-negative, or other bacteria; or fungi. The survival of pathogens was determined as follows: From every publication, the data were extracted and clustered according to pathogen group (non-enveloped and enveloped viruses; gram-positive, gram-negative, and other bacteria; and fungi), surface, temperature, and relative humidity (RH). We included only studies that reported infectivity of the examined virus—and not just polymerase chain reaction (PCR) positivity.

## 3. Results

### 3.1. Viruses

Infections of viruses can lead to a broad range of clinical courses—from asymptomatic infections to severe diseases with a possible deadly outcome. In the following section, we report the viral survival times on multiple surfaces. A summary of the results can be obtained from Table 1 and Table 2.

### 3.2. DNA Viruses

#### 3.2.1. *Adenoviridae*

The family *Adenoviridae* comprises nonenveloped viruses with an icosahedral nucleocapsid containing a double stranded DNA genome, causing a wide range of illnesses, from mild respiratory tract infections or conjunctivitis to life-threatening multi-organ disease in patients with a weakened immune system [17]. Importantly, in addition to aerosols, fomites (environmental surfaces) play an important role for the transmission of adenoviruses. Due to the relevance of transmission via surfaces, extensive research has been performed regarding the survival on surfaces: Mahl et al. examined the survival of adenovirus type 2 on glass, vinyl asbestos floor tiles, ceramic tiles and stainless steel at 37 °C, two different light conditions (“light” and “dark”) as well as 93% and 7% RH [21]. Although the authors did not observe large differences between the fomites, the virus survived longer than 8 weeks at 7% RH with a 2-log reduction in infectivity titer. In contrast, at 93% RH inactivation was already observed after 1 h/3 weeks (“light”/“dark”, respectively). Importantly, despite a 4-log titer reduction, on glass the virus survived for more than 12 weeks at 37 °C and 25 °C at low humidity [21]. Gordon et al. showed that some adenovirus strains can survive on plastic and aluminum for up to 49 days, using six different strains. Although, all six strains were still detectable after 49 days on plastic, some strains survived notably better than others. On aluminum, the virus strains could be recovered between 28 to 49 days after inoculation. Especially the strain AD19 ATCC persisted the longest on aluminum among the tested strains. On plastic, the differences between the strains were not significant [18]. Next to four other enteric viruses, Abad et al. examined the survival human adenovirus type 40 on aluminum, china, latex, and paper under three environmental conditions (4 °C + 90% RH, 20 °C + 85% RH, 20 °C + 50% RH). For each condition, the virus was suspended either in phosphate-buffered saline (PBS) or in 20% fecal suspension (FS). Depending on the conditions tested, ADV survival ranged between seven and 60 days with at least a 4-log reduction of viral titers. Interestingly, the two different suspensions displayed a surface-dependent impact on virus stability—on aluminum, china and latex, FS had a protective effect; on paper, the virus survived longer in PBS. The virus survived significantly longer at 4 °C than at 20 °C [19]. Rabenau et al. showed that adenovirus type 3 can survive on plastic at room temperature (RT) at least 9 days, accompanied by a 2–3-log reduction of infectious viral titers. The presence or absence of 10% fetal calf serum (FCS) did not significantly alter virus survival [20].

In conclusion, the survival of adenoviruses ranges between 9 days and more than 12 weeks. This range may be explained by the examined subtypes, surfaces, environmental conditions, and investigation period (Table 1).

#### 3.2.2. *Papillomaviridae*

*Papillomaviridae* are a large family of non-enveloped DNA viruses, which are highly host- and tissue-tropic, typically infecting the skin or mucosal epithelium. Using pseudotyped human papillomavirus type 16 (HPV-16), Roden et al. observed virus persistence for up to 7 days at RT [22].

#### 3.2.3. *Herpesviridae*

*Herpesviridae* are a large family of enveloped DNA viruses, which frequently remain in a latent state and thus cause unapparent infections, which may result in opportunistic infections within immunocompromised individuals. In particular, Herpes simplex virus 1 and 2 (HSV-1 and HSV-2), varicella zoster virus, Epstein–Barr virus, and cytomegalovirus are widespread among humans [64,65,66].

##### Herpes Simplex Virus (HSV)

HSV was detectable on plastic between 2 days and 3 days when kept at RT, which was reported by Firquet et al. and Rabenau et al. [20,23]. The latter study additionally found that an addition of 10% fetal calf serum (FCS) could extend the survival time up to 6 days. A study by Nerurkar et al. showed that an increased temperature (37–40 °C) and a more humid environment led to a decreased viral persistence. The virus was only infective for 4.5 h at the given temperature [24]. In contrast to the previous studies, Mahl et al. examined the survival of HSV on glass slides at RT and 37 °C. At RT, the virus was detectable for 1 days at 96% and 55% RH, while at 37 °C and the same RH, the virus survived only for 4 h. However, at 3% and 7% RH, the virus survived for more than eight weeks at both temperatures [21]. This sorts well with the findings on plastic: A higher temperature inactivates the virus quicker. Furthermore, RH seems to correlate negatively with the survival time resulting in variable observations regarding virus persistence (Table 1).

##### Cytomegalovirus (CMV)

With only two studies available, research about CMV stability is limited. One study tested the survival of CMV acquired by infected infants on several surfaces and observed survival on plexiglass for up to 8 h and on cotton blankets for up to 2 h at RT [25]. In a second study, the author showed that at RT, CMV could be recovered from latex gloves after 4 h [26].

#### 3.2.4. *Poxviridae*

##### Vaccinia Virus

Overall, vaccinia virus is extraordinary stable on surfaces: both Mahl et al. [21] and Wood et al. [27] examined the survival of the virus on several surfaces and environmental conditions and observed that the virus persisted for up to eight weeks. The examined surfaces included glass, galvanized steel, cinder, and industrial carpet. In general, higher temperatures and RH were associated with reduced virus survival. Furthermore, at RT the persistence of Vaccinia virus was generally longer on hard, nonporous materials (glass, galvanized steel) [27].

*Poxviridae* viral particles are a family of enveloped DNA virions, including the highly pathogenic Variola virus causing smallpox.

### 3.3. RNA Viruses

#### 3.3.1. *Reoviridae*

*Reoviridae* are a diverse family of non-enveloped RNA viruses that mainly cause mild or subclinical symptoms upon infection. However, rotavirus infections are among the leading causes of severe infectious diarrhea in children and have been frequently associated to fomite-mediated transmission [67].

The survival of human rotavirus (Wa strain) was examined during a study of Sattar et al., who tested the persistence of the virus on several porous (poster card and cloth) and non-porous (glass, stainless steel, vinyl, and smooth plastic) surfaces at different environmental conditions [28]. On all non-porous materials, the virus remained infectious even after 10 days at RT and medium/low RH (50 ± 5%/25 ± 5%, respectively), while at RT and high RH (85 ± 5%) the virus was almost completely inactivated after 48 h. The virus could also survive for longer than 10 days on stainless steel at 4 °C with a significantly lower inactivation rate than at RT. On a poster card, the virus was not detectable after desiccation, while on cloth at infectious virus could be recovered after 2 days up to 10 days at RT and 4 °C, respectively, each at ambient RH.

To examine survival on counter tops, Keswick et al. suspended the virus either in distilled water or in a solution of water containing 10% rotavirus-negative stool [29]. After desiccation, virus could be detected for up to 30 min and for up to 60 min, respectively [29]. According to Abad et al., rotavirus could survive on aluminum, china, latex, and paper for more than 60 days, regardless of the examined environmental condition or whether FS was added or not [19]. The loss of titer was less than three orders of magnitude in each of the conditions [19].

#### 3.3.2. *Picornaviridae*

*Picornaviridae* are a large family of nonenveloped RNA viruses, including some of the most common and important human pathogens which are responsible for a variety of human disease, including poliomyelitis, the common cold, hepatitis A, and enteric diseases [68].

##### Coxsackie Virus

The survival time of Coxsackie virus was reported to be at least two weeks: a study of Firquet et al. showed that at RT coxsackie virus B4 survived on plastic for five weeks, using an initial titer of roughly 10^7^ TCID_50_/mL [23]. In contrast, Mahl et al., observed inactivation of coxsackie virus B3 after three weeks on glass at 25 °C and 37 °C at high, medium and low RH [21].

##### Echovirus

On cellulose ester membrane filters Mocé-Llivina et al. bserved that Echovirus 6 was more persistent at high RH levels than at low RH levels [31]. Importantly, at high RH the virus survived for more than 7 days at RT and 4 °C without significant reduction in infectivity, while at moderate RH a 2.5-log reduction was observed at both temperatures over time [31].

##### Poliovirus

Together with other enteric viruses, Abad et al. tested the survival of Poliovirus 1 on aluminium, china, latex and paper under different environmental conditions (4 °C + 90% RH, 20 °C + 85% RH and 20 °C + 50% RH) and additionally in a PBS or fecal suspension (FS) [19]. Virus survival was observed from 3 days up to 60 days (china + 4 °C + 90% RH) [19]. Likewise, Mocé-Llivina et al. observed higher virus survival of poliovirus 1 on cellulose ester membrane filters at 4 °C as compared to RT [31]. Despite a significant reduction in viral infectivity, Tamrakar et al. observed Poliovirus 1 survival on steel, plastic and cotton after three weeks, when kept at RT and 40–60% RH [32]. Mbithi et al. compared Poliovirus 1 with Hepatitis A virus and showed, that poliovirus was inactivated on stainless steel after 24 h and 6 h at 20 °C + 95 ± 5% RH and 20 °C + 25 ± 5% RH, respectively [33]. Mahl et al. studied the survival of Poliovirus 2 on glass slides at several temperatures (25 °C and 37 °C) and RH (high, moderate and low) [21]. At 37 °C, the virus survived for eight weeks at low humidity and for one day at high and moderate RH—the higher titer was found at a high RH. At RT, the virus survived for eight weeks at low RH, for four weeks at high RH, and for two weeks at moderate RH [21].

In summary, the survival time of poliovirus ranges between 1 day and 60 days, which shows that virus stability is highly susceptible to environmental conditions (Table 2).

##### Rhinovirus

Human rhinoviruses (HRV) are non-enveloped, positive single strand RNA viruses, classified into three species (A–C). They mainly cause respiratory tract infections, like the common cold, but they were also reported to cause otitis media. Predisposed groups for a critical infection are immunocompromised and elderly adults; a severe bronchiolitis can occur in infants. HRV can exacerbate a preexisting chronic lung condition as asthma bronchiale, chronic obstructive pulmonary disease, or cystic fibrosis. Transmission happens via aerosols, direct contact or fomites [69,70,71]. A study of Syed et al. suspended rhinovirus type 14 in either tryptose phosphate broth (TPB), bovine mucin or human nasal discharge to inoculate stainless steel disks. In nasal discharge, the virus persisted for up to 8 h. In contrast, in TPB and bovine mucin the virus was still detectable after 24 h. The authors further observed a high RH had a protective effect on virus survival [34].

##### Hepatitis A-Virus

The hepatitis A-virus (HAV) is a non-enveloped, positive single strand RNA virus. Virus transmission mainly occurs via the fecal-oral route [72,73]. Mbithi et al. examined the survival of HAV on stainless steel at 5 °C, 20 °C, and 35 °C. At 5 °C, the virus persisted for more than 4 h regardless of the RH. At 20°C, HAV was still detectable after 14 days and at 37 °C, the virus survived for 4 h—except at 95 ± 5% RH, where it was inactivated after 4 h [33]. HAV survived for more than 60 days on aluminum, china, and latex and for more than 30 days on paper at all tested environmental conditions as shown by Abad et al. [19]. During a study from Kim et al., HAV persisted on wood and stainless steel for more than 30 days at different environmental conditions [35]. Overall, throughout the different studies higher RH levels were associated with shorter virus survival.

#### 3.3.3. *Caliciviridae*

*Caliciviridae* are a family of positive single strand RNA viruses without a lipid envelope. The most prominent member of this family is the human norovirus, a common causative agent of non-bacterial acute gastroenteritis. Human noroviruses are transmitted fecal–orally and due to its high infectivity tends to create outbreaks—especially in healthcare facilities. Since the cultivation in a cell culture system remains difficult, several surrogates have been established [74,75,76].

##### Feline Calicivirus (FCV)

One of the established surrogates is the feline calicivirus: D’Souza et al. examined the survival of FCV on Formica^®^, ceramics and stainless steel at RT and 75–88% RH [36]. The virus could be recovered from all surfaces after 7 days, with a 6–7-log reduction in infectious titer [36]. The survival times observed by Clay et al. were shorter than reported by D’Souza et al.: The authors studied the survival of FCV on uncommon surfaces at RT. The virus was undetectable on keyboard keys and brass after 8–12 h, on computer mouse and telephone wire after 24–48 h, and on telephone buttons and telephone receiver after 48–72 h [37]. Buckley et al. observed FCV survival at RT and 30% RH on wool for 15 days and on nylon and glass for up to 7 days. The persistence of FCV at 70% RH was shorter on all surfaces as compared to 30% RH [40].

##### Murine Norovirus

Another established surrogate is the murine norovirus (MNV). Additionally, to FCV, Buckley et al. further examined the persistence of MNV on wool, nylon and glass [40]. At RT and 30% RH, the virus survived up to 15 days on wool and under 15 days on nylon and glass. At 70% RH, the virus survival was lower for all examined conditions [40]. Warnes et al. studied the effect of copper on virus inactivation on dry and wet fomites. Using stainless steel as a control and viral persistence over the examined 120 min was observed. Virus survival was further observed to be proportional to copper content. On pure copper, MNV was inactivated after 30 min and 5 min on wet and dry fomite, respectively [39]. A slightly longer persistence was shown by Kim et al. The authors showed that MNV persisted on wood for longer than 30 days and on stainless steel for up to 20 days [35].

##### Tulane virus

To test Tulane virus as a surrogate for Caliciviridae, Arthur et al. observed Tulane virus survival on acrylic-based solid and stainless steel, for at least 14 days [38]. The reduction in infectious titer on stainless steel was significantly higher than that on acrylic-based solid—with roughly 1-log reduction in titer after 14 days [38]. These results are in accordance with observations of the other two surrogates (Table 2).

#### 3.3.4. *Flaviviridae*

##### Hepatitis C-Virus (HCV)

In two studies the survival of HCV on surfaces is reported: Paintsil et al. examined the survival of low-titer (10^4^ infectious units/mL) and high-titer (10^6^ infectious units/mL) HCV on 24-well-plates (plastic). Low-titer HCV was recovered after 6 weeks at 4 °C and 22 °C, while being inactivated after 7 days at 37 °C. High titer HCV survived for at least three weeks at 4 °C and 22 °C with almost no loss of titer, while after 21 days at 37 °C the virus was inactivated [41]. In contrast to the previous study Doerrbecker et al. found that the HCV strain Jc1 survived on stainless steel at RT for 5 days, when suspended in human serum and for more than 7 days without being suspended in serum [42]. In the latter case, there was a 3-log reduction visible.

#### 3.3.5. *Orthomyxoviridae*

The family of *Orthomyxoviridae* includes the influenza A–C viruses, which contain a segmented single-strand RNA with a negative orientation and a lipid envelope. While Influenza A viruses are classified according to their haemagglutinin and neuramidase proteins, Influenza B viruses are separated into two major lineages (“Victoria” and “Yamagata”). Influenza viruses cause the common flu, but can also cause complications, like the primary influenza-associated pneumonia. The ability to rapidly adapt to changing environmental conditions via antigen shift and antigen drift leads to a tendency to form highly infective strains. Transmission mainly occurs by aerosols, but also via inanimate objects [77,78,79,80].

##### Influenza A Virus

Since Influenza A virus plays an important clinical role, considerable research has been conducted. Bean et al. showed that Influenza A virus survived on stainless steel for at least 48 h, with a 3-log reduction. On plastic, tissues and magazines the virus survived for 48 h and on handkerchiefs and pajamas for 24 h. The environmental conditions were 27.8–28.3 °C and 35–40% RH [43]. A shorter survival time was reported by Greatorex et al. who also studied the survival of Influenza A virus on several surfaces, and observed a 3.9-log reduction in infectivity on stainless steel after 9 h [44]. Likewise, the virus was viable on plastic after 9 h, with a slightly greater reduction (4.2-log). On silver containing cloth, soft toys and light switch material, Influenza A virus survived for more than 4 h and less than 9 h. And on J-cloth^®^, window glass, telephone handset, kitchen work surface, computer keyboard, aluminum, pine, varnished and unvarnished oak, the virus was not detectable after 4 h. Greatorex et al. performed all experiments at 17–21 °C and 23–24% RH and with an initial titer of 3 × 10^5^ PFU/mL [44]. Influenza A virus (strain A/Mexico/4108/2009 (H1N1)) showed shorter survival times of infectious virus on plastic and steel compared to MERS-CoV: No infectious virus was detected after 8 h, regardless the tested environmental conditions (20 °C + 40% RH, 30 °C + 30% RH and 30 °C + 80% RH), van Doremalen et al. showed [56]. In contrast, Firquet et al. observed infectious Influenza A virus (strain H1N1 A/PR/8/34) on plastic petri dishes was inactivated after 5 days at RT [23]. Perry et al. observed that two strains of Influenza A virus remained infective on steel after 7 days under various environmental conditions (18 °C + 20% RH, 25 °C + 20% RH, 18 °C + 35% RH, 25 °C + 35% RH, 18 °C + 55% RH, 25 °C + 55% RH) [45]. To examine the influence of light on the survival of Influenza A virus, Thompson et al. surveyed five different strains on three materials (cotton, microfiber and stainless steel) in light and dark environment [46]. In a dark environment, all strains survived on stainless steel for two weeks. On microfiber, the range of survival time was between less than an hour and one week. On cotton, one strain survived for one week and the other strains for 24 h. The survival times in a light environment were comparable to the ones in the dark environment setting. All experiments were performed at RT and 10–50% RH [46].

In summary, the survival time of Influenza A virus has a broad range from a few hours to several weeks (Table 2). An explanation could be the differences in environmental conditions, tested surfaces, viral subtypes, and initial titers.

##### Influenza B Virus

In contrast to Influenza A virus, Influenza B virus lacks extended research: Bean et al. examined the viability of Influenza B virus on several surfaces after drying. While the virus was inactivated after 48 h on plastic and stainless steel, virus survival on pajamas for 12 h was observed. On handkerchiefs, tissues, and magazines, viral persistence for 8 h was reported. The experiments were performed at 26.7–28.9 °C and 55–56% RH [43].

#### 3.3.6. *Paramyxoviridae*

##### Parainfluenza Virus

The survival of parainfluenza virus 2 on several non-absorptive and absorptive surfaces at RT was examined by Brady et al. using an initial titer of 1.5 × 10³ TCID_50_/mL. On stainless steel, the virus survived between 2 h and 4 h, when dried, and more than 10 h as a droplet. On Formica^®^, dried virus was inactivated after 1 h and remained infectious after 8 h as droplet. On a lab coat and on facial tissues, there was no titer reduction visible after 4 h, while on hospital gown, the virus was inactivated after 0.5–4 h, depending on the pretreatment [47].

##### Henipavirus

Hendra virus and Nipah virus have a comparable susceptibility to desiccation, as observed by Fogarty et al. [48]. When dried on non-porous polystyrene, both Hendra and Nipah virus survived for less than 15 min at 37 °C. At 22 °C, Hendra virus survived for less than 60 min, and Nipah virus was still detectable after 60 min showing a 2-log reduction [48].

##### Respiratory Syncytial Virus (RSV)

To examine RSV survival on surfaces, Hall et al. suspended RSV in nasal secretions of healthy adults and infants with a lower respiratory tract infection and applied it to surfaces [49]. All experiments were performed at RT and 35–50% RH and with an initial titer of 10^5^–10^6^ TCID_50_/mL. On countertops, the virus was no longer detectable after 7–8 h. On paper tissues, cloth and gown, the virus survived for less than 1 h, less than 1–2.5 h and less than 2–5.5 h, respectively [49].

#### 3.3.7. *Filoviridae*

##### Ebolavirus

The Ebola viruses is enveloped and encodes its genome in the form of single-stranded negative-sense RNA. Research about the survival of Ebola virus on surfaces is limited. Yet, in one study of Westhoff Smith et al., the authors observed survival of Ebola virus in syringe needles between 16 days and 32 days, when suspended in Dulbecco’s modified Eagle’s medium (DMEM), while upon suspension in whole blood the persistence of viral particles up to 48 days was observed [50]. The examined environmental conditions were RT and 55% RH and the initial titer was 10^6^ PFU/mL. Upon suspension of an initial titer of 10^7^ PFU/mL in whole blood, no infectious virus could be recovered from in syringe needles after 32 days at 32 °C/80% RH, while at RT/55% RH, only a one log reduction was observed. On banknotes, the virus survived for more than 4 days (21 °C, 55% RH) and 2 days (32 °C, 80% RH). Both experiments had an initial titer of 10^7^ PFU/mL [50].

#### 3.3.8. *Coronaviridae*

*Coronaviridae* consist of positive single strand RNA viruses with an envelope, contributing to its name (from lat. corona—crown). There are seven human pathogenic strains of Coronaviruses (CoV)- 4 endemic strain (229E, OC43, NL63, and HKU1) and 3 epidemic strains (Severe acute respiratory syndrome coronavirus (SARS-1-CoV), Middle East respiratory syndrome coronavirus (MERS-CoV), severe acute respiratory syndrome coronavirus type 2 (SARS-CoV-2). While the endemic strains mainly cause a mild respiratory infection—like a common cold—the epidemic strains cause a viral pneumonia and gastrointestinal symptoms as diarrhea or vomiting. SARS-CoV-2 is responsible for the 2020 COVID-19 pandemic. The mode of transmission is mainly by respiratory droplets and aerosols, but a certain contribution of contact transmission, e.g., via fomites seems likely [81,82,83,84].

##### HCoV-229E

HCoV-229E is reported to survive below 6 h on latex gloves and below 12 h on aluminum and sterile sponges at RT and 55–70% RH using a titer of 5.5 × 10^5^ TCID_50_/mL [51]. In contrast, using an initial titer was >10^6^ TCID_50_/mL, Rabenau et al. observed that HCoV-229E could persisted on plastic for even 50 h at RT [20]. According to Warnes et al., infectivity of HCoV-229E was detectable for more than 5 days on Teflon^®^, PVC, ceramic, stainless steel, and glass—although showing approximately a 2-log reduction in titer [85]. On silicon rubber, the virus remained infective for more than 3 days but less than 5 days. On surfaces with a high content of copper, the virus was inactivated at a much faster scale (minutes–hours). The experiments were performed at RT and moderate RH using an initial titer of 5 × 10^4^ PFU/mL [85].

##### HCoV-OC43

The study of Sizun et al. also involved HCoV-OC43, which survived shorter on different surfaces as compared to HCoV-229E—infectivity was lost less than one (latex gloves, sterile sponges) or 3 h (aluminum) [51].

##### SARS-CoV-1

SARS-CoV-1 could, according to Rabenau et al., survive on plastic and at RT for up to 8 days [20]. While Rabenau et al. used an initial titer of ~5 × 10^6^ TCID_50_/mL, Chan et al. used a titer of 10^7^ TCID_50_/mL and observed approximately a 5-log reduction of viral infectivity on plastic after 13 days at RT and a RH of 40–50% [52]. The virus seemed to have a shorter survival time on non-porous material: Lai et al. examined the survival of SARS-CoV-1 on paper, disposable and cotton gown and observed virus inactivation after 24 h on paper and cotton gown, and after 2 days on disposable gown using an initial titer of 10^6^ TCID_50_/mL, while at lower titers shorter survival times were observed [54]. In contrast to Rabenau et al. van Doremalen et al. compared SARS-CoV-1 and -2 (see below) and observed that at RT and 40% RH SARS-CoV-1 was inactivated after 24 h on cardboard, after 8 h on copper, and after 72 h on stainless steel and plastic [20,53]. To test two surrogates of SARS-CoV-1, Casanova et al. examined transmissible gastroenteritis virus (TGEV) and mouse hepatitis virus (MHV) on stainless steel. Both viruses were most stable at 4 °C and after 28 days less than 2-log reduction of the initial titer was observed. Upon higher temperatures, the viruses were inactivated more rapidly—at 40 °C and 80% RH, both viruses survived only for 6 h. While low temperatures and RH had a protective effect on virus survival, the relationship between inactivation and RH was not monotonic, and a greater protective effect at low RH (20%) and high RH (80%) than at moderate RH (50%) was observed [55].

##### MERS-CoV

MERS-CoV was inactivated on plastic and stainless steel after 48 h, 24 h, and 8 h at 20 °C/40% RH, 30 °C/30% RH and 30 °C/80% RH, respectively. The initial titer, used by van Doremalen et al., was 10^6^ TCID_50_/mL [56].

##### SARS-CoV-2

As described above, van Doremalen examined the survival of SARS-CoV-1 and -2. The authors could not observe viable SARS-CoV-2 on copper after 4 h, on cardboard after 24 h, and on stainless steel and plastic after 72 h [53]. Furthermore, Chin et al. studied the persistence of SARS-CoV-2 at RT on several surfaces, those being paper, tissue paper, wood, cloth, glass, banknotes, stainless steel, plastic, and the inner and outer layer of a surgical mask [57]. While on paper and tissue paper, no infectious virus was detectable after 3 h, on wood and cloth, the virus persisted for 1 day. Infectious virus was detectable for 2 days on glass and banknotes and for 4 days on stainless steel, plastic, and the inner mask layer. Furthermore, there was still infectious particles detectable after 7 days on the outer layer of a surgical mask. Chin et al. further discovered, that SARS-CoV-2 showed a biphasic decay, with a short half-time in the first 3 h and a longer half-time after that [57]. According with the previous papers, Kratzel et al. found a survival on stainless steel between 17 days and more than 30 days, depending on the temperature [58]. Interestingly, a higher temperature did not lead to an increased viral decay—like that shown before on other coronaviruses and other viral pathogens.

#### 3.3.9. *Astroviridae*

The survival of astroviruses was studied by Abad et al. on china as well as paper. The conditions tested were 20 °C and 4 °C (both 90 ± 5% RH), and suspension in PBS and 20% fecal suspension (FS). On china, the virus could persist for 7 days at 20 °C and 60 days at 4 °C. In contrast, the virus survived on paper for at least 90 days at 4 °C with a 4-log reduction, regardless if suspended in PBS or FS. At 20 °C, the virus remained infective for up to 60 days upon suspension in PBS, and for 7 days upon suspension in FS [59].

#### 3.3.10. *Retroviridae*

##### Human Immunodeficiency Virus (HIV)

Although a transmission of HIV via fomites is unlikely, two studies examined the persistence of the lentivirus on surfaces: Barre-Sinoussi et al. showed that HIV-1 persisted on petri dishes of unknown material for at least 7 days at RT [60]. A similar result was found by Tjøtta et al., who examined the survival of HIV-1 on glass slides and observed that the virus persisted for 5 days on the slides at RT using an initial titer of 9.1 × 10^6^ particles/mL [61].

#### 3.3.11. *Hepadnaviridae*

##### Hepatitis B Virus (HBV)

After its discovery in the 1970s, two studies have been performed regarding the survival of HBV on surfaces: Bond et al. showed, that HBV was still infectious after drying in silanised tubes for one week at RT and 42% RH [62]. At the same environmental conditions, a study by Favero et al. showed that there was virtually no loss antigenic activity of HB Ag-positive blood after 14 days from stainless steel and cotton swabs [63].

### 3.4. Bacteria

Infections with bacteria are causing a broad range of human diseases. Many of the infections caused by human-pathogenic bacteria are linked to hospitalization of patients. Several severe and life-threatening infections are caused by nosocomial-acquired bacterial infections. Among these bacteria are species of the genera *Staphylococcus*, *Enterococcus*, *Klebsiella*, *Stenotrophomonas*, and *Pseudomonas*. All of these species have been reported to be isolated from various abiotic surfaces and bacterial survival analyses on distinct surfaces have been published.

In the following section, we will summarize the state of art on survival properties of human pathogenic bacteria on numerous inanimate surfaces. A summary is also given in Table 3, Table 4 and Table 5.

#### 3.4.1. Gram-Positive Bacteria

Infections with gram-positive bacteria are regularly reported. Among the most prominent gram-positive human pathogens are staphylococci.

##### Staphylococci

One of the most relevant human pathogenic gram-positive bacteria is *Staphylococcus aureus*. *S. aureus* is a well-studied human pathogen commonly causing both, community- and hospital-acquired infections. *S. aureus* is known to be one leading causative microorganism for bacteraemia and sepsis worldwide [153]. Methicillin-resistant *S. aureus* (MRSA), resistant to β-lactam antibiotics, are regularly causing difficult-to-treat infections. Infections with MRSA are associated with increased mortality rate in comparison to infections with methicillin-susceptible *S. aureus* (MSSA) [154]. Due to the link between *S. aureus* infection and hospitalization, the survival of *S. aureus* on abiotic surfaces has been studied to a significant extend.

Scott et al. studied the survival of several bacteria, among them *S. aureus*, on laminate and j-cloth^®^. In this study, they distinguished between wild-type (WT) and laboratory *S. aureus* strains as well as between a clean and soiled condition (simulated by tryptone soya broth). While on laminate both strains were still viable after 24 h regardless of the applied conditions, growth analysis on j-cloth^®^ showed viable cells of the laboratory strain after 48 h whereas the WT strain was inactivated after 24 h [104].

Kampf et al. could demonstrate that *S. aureus* was able to survive on a polymer for 7 days [105]. On that time point, *S. aureus* was almost completely inactivated. That relatively short survival in contrast to other studies can be explained with the temperature being higher (37 °C) as those in the other studies [105].

Several studies compared MRSA strains with MSSA strains on their survival capabilities. On cotton lint, both MRSA and MSSA have been shown to persist for more than 25 days with a ~1.5-log reduction in titer at that time point [96]. Additionally, Wagenvoortand and Penders examined the persistence of MRSA and MSSA on plastic and analyzed the effect of dust on both. In contrast to the study by Hirai, a clear difference in survival of MRSA and MSSA was detectable: while MRSA could persist on plastic for up to 175 days with a steady ongoing decline, MSSA was only detectable for 28 days, rapidly declining. The authors explained those differences with the studied MRSA strain (phage-type III-29), which potentially could have better survival properties than other MRSA strains. In addition, dust had a negative effect on the survival of both MRSA and MSSA [106]. In a further study, a survival comparison of two MRSA outbreak strains (III-29, III-215) with three sporadic MRSA strains (“untypable”/UT, Z-69, III-152) on plastic has been done. The outbreak strains were viable for up to 318 days, while the sporadic strains survived significantly shorter (287 days or shorter). Also, the presence of dust had a negative impact on survival of sporadic strains but not on outbreak strains [107].

Neely and Maley studied the survival of MRSA and MSSA on several cloths and polyethylene surfaces. While on cotton, the bacteria have been shown to survive for up to 21 days, living cells could be detected for up to 56 days on polyester and on polyethylene both strains were still detectable after 51 days. MSSA was additionally detectable after 91 days on polyethylene. However, the investigation period was extended in comparison to MRSA and thus no trustworthy comparison could be made [91].

To study the effects of copper on MRSA survival, Noyce et al. compared the survival of three strains of MRSA on copper, stainless steel and brass. All strains were inactivated after 90 min on copper, whereas there was almost no loss in titer after 6 h on stainless steel. Survival on brass, which consists to 80% of copper, showed that two of the three strains were inactivated on brass after 270 min, and only one strain was still detectable after 6 h. However, this strain showed almost a 2-log reduction in titer. Lowering the temperature from 22 °C to 4 °C has been detected to prolong the bacterial decay [109]. According to Webster et al., *S. aureus* survived on Formica^®^, stainless steel and a drip stand for 9, 10, and 3 days, respectively. However, the initial titer was not specified [110]. An aggregate of five different MRSA strains survived for more than 6 weeks on stainless steel as shown by Otter et al. [87]. Although being still viable after 6 weeks, there was a 5.5-log reduction in *S. aureus* titer visible [87]. However, single strains showed persistence from less than four week [87]. A study on several mutations of *S. aureus*, including the strain SH1000 as a reference, revealed a persistence of SH1000 on dry plastic surfaces (polypropylene) for more than 1097 days [108]. *S. aureus* was able to survive on glass coverslips for 15–25 days, when kept in distilled water. Replacement of water with bovine serum albumin caused an even longer *S. aureus* survival on glass [111]. An additional comparison of ambient medium showed that *S. aureus* was able to survive on ceramic floor, synthetic fiber, cotton and mattress for more than eight weeks, when kept in distilled sterilized water, urine, blood, or saliva. In all conditions, there was a maximum of 2-log reduction in titer after eight weeks [97].

Even though most studies worked with *S. aureus*, survivals on inanimate surfaces of other clinically relevant staphylococci as *Staphylococcus epidermidis* have been performed as well. *S. epidermidis* is a skin commensal. However, due to its occurrence and survival on medical devices, *S. epidermidis* is described as a significant nosocomial bacterial agent [155]. It has been shown that *S. epidermidis* survived on cotton lint for more than 25 days and on glass for more than 7 h with almost no loss in titer [96]. Neely et al. studied the survival of not-further defined coagulase-negative staphylococci on several cloths and polyethylene. While the bacteria survived on cloths from 6 to 28 days, they were partially still viable after more than 90 days on polyethylene [91].

The survival capabilities of staphylococci demonstrated in numerous studies showed clearly the persistence of the bacteria, particularly of *S. aureus*, to be surface dependent, ranging from hours to weeks and even years (Table 3).

##### Streptococci

Bacteria of the genus *Streptococcus* have been reported to be responsible for several health issues. Among the most prominent pathogens within this genus are *Streptococcus pyogenes*, frequently causing pharyngitis and deep tissue infections and *S. pneumoniae* known to cause cases of bacterial pneumonia in humans [156]. Various studies reported the survival capabilities of streptococci on different inanimate surfaces. Persistence of *S. pyogenes* has been described to range between 2 h and four months in a surface-dependent manner. On glass, metal and plastic surfaces time of survival has been detected to range from 2 h to 88 h. The same time period has been found for wood and latex, however, the loss in viability was much higher on the latter surfaces compared to glass [116]. In a study survival on food and food contact surfaces it has been detected that *S. pyogenes* survives for more than 24 h on the surface of refrigerated tomatoes. After 2 h, *S. pyogenes* has still been detected viable on the surface of plastic cups, ceramic plates, and stainless steels at RT [115]. In a study on musician instruments like reeds and clarinets, *S. pyogenes* has been examined on its survival. It has been shown that the bacteria survive for 24 h, even when the clarinet is simulated to play [117]. Both, *S. pyogenes* and *S. pneumoniae* are capable to survive on plastic surfaces for several days to months. Biofilms of *S. pyogenes* survive for more than four months while these from *S. pneumoniae* persist for one month [114].

*Streptococcus salivarius* is a commonly harmless colonizer of the human oral cavity but linked to more harmful infections (e.g., endocarditis) in immunosuppressed patients [156]. It showed survival durations comparable to *S. pyogenes* on glass, metal, plastics, wood, and latex with between <2 and 88 h in dependence on the ambient liquid medium [116]. *Streptococcus equi* belong to the Lancefield group C streptococci and is widely recognized as an animal pathogen. However, also association with human diseases are done [156]. Its survival has been examined in two studies. Both revealed a survival from less than one day up to 13 days on wood, clothing, metal and rubber surfaces [112,113]. Next to the type of surface, also effects of sunlight [112], temperature [115], medium [116] and bacterial life form [114] have been demonstrated. While an increase of temperature from 5 °C to 21 °C resulted in survival times rising from less than 2 h to more than 24 h for *S. pyogenes* [115] and sunlight causing a decrease in the survival time of *S. equi*, rain did not affect the survival time of *S. equi* [112]. The life form of the bacteria within a biofilm also affected survival capacity in a beneficial way. For both, *S. pyogenes* and *S. pneumoniae* this effect has been shown on plastic surfaces [114]. Each of the above-mentioned streptococci isolates showed a species and surface dependent survival on the tested inanimate surfaces.

##### Enterococci

Besides staphylococci, also enterococci have been demonstrated to survive on several abiotic surfaces. Enterococci are opportunistic pathogens, being a known leading cause of nosocomial infections. Particularly two enterococci species are known to cause infections in humans, among them bacteraemia, endocarditis, and urinary tract infections. These species are *Enterococcus faecalis* and *Enterococcus faecium*. In the past, the emergence of vancomycin-resistant enterococci (VRE) increased, which is connected to increased mortality [157].

Among others, a study of Hirai, published in 1991, stated that *E. faecalis* was still detectable after one day on cotton lint [96]. In contrast, much longer survival has been demonstrated in a study of Neely and Maley in which the authors showed that *E. faecalis* could persist on cotton, terry (100% cotton) and a cotton–polyester blend for up to 33 days. Further they detected that it persisted for more than 90 days on polyester and polyethylene, without a significant difference between VREs and vancomycin-sensitive enterococci (VSE) [91]. In the work from Esteves et al. the long persistence on cotton could been proven as *E. faecalis* was still viable on cotton after eight weeks. Additionally, they reported that *E. faecalis* survived on synthetic fiber for two weeks, on ceramic floor for three weeks and, on mattress for four weeks when kept in distilled water [97]. According to Noskin et al, *E. faecalis* was able to survive on countertops for 5 days. This comparatively short time could be explained with the initial titer of 10^4^ colony-forming units (CFU) [95]. Bale et al. revealed that *E. faecalis* survived for more than 77 days on glass slides [93]. Wendt et al. compared the survivability on polyvinyl chloride of *E. faecalis* and *E. faecium* as well as of VREs and VSE. *E. faecalis* could persist for between one week and four months. Moreover, the difference in survival for *E. faecalis* and *E. faecium* and for VRE and VSE was not significant [94].

To study the survivability of VRE, Otter and French examined the persistence of two strains of *E. faecium* and one strain of *E. faecalis*. The aggregate of these three strains was able to survive for 6 weeks on stainless steel with a reduction of three log [87]. Bale et al. showed that *E. faecium* could persist on glass slides for more than 77 days. There was no significant difference between *E. faecium* and *E. faecalis* [93].

Noskin et al. examined the survival of *E. faecium* on countertops, where it was viable for 7 days. The initial titer was comparatively low with 10^4^ CFU [95]. In contrast to the other studies, where the survival of *E. faecalis* and *E. faecium* was relatively similar, Neely and Maley showed that *E. faecium* could survive for more than 80 days on each of the tested materials in a strain-dependent manner (cotton, terry, blend, polyester, polyethylene); thus, *E. faecium* has higher survival capabilities than *E. faecalis* [91].

In addition to the most clinical relevant enterococci, also studies on less well-known enterococci as *E. gallinarum* and *E. casseliflavus* have been published [91]. Among others, Neely and Maley showed that *E. gallinarum* can persist on cotton, terry and cotton-polyester blend for 28, 34, and 34 days, respectively. On polyester and polyethylene, *E. gallinarum* was found to be even viable after 90 days [91]. *E. casseliflavus* survival studies revealed comparable viability over time, while on cotton, terry, and blend, it survived for 15, 28, and 15 days, it was still viable after 90 days on polyester and polyethylene [91]. Jawad et al. could show that not further specified enterococci were able to survive on glass for roughly 3 days. The suspension in BSA increased the survival significantly—to roughly 45 days [111]. Each of the reported enterococci species showed a species and surface dependent survival on various inanimate surfaces (Table 3).

##### *Clostridioides difficile* 

*Clostridioides difficile,* another human relevant gram-positive bacterial pathogen, is frequently causing healthcare-associated infections. Thus, studies on the transmission of *C. difficile* via hospital surfaces have been published. It has been demonstrated that *C. difficile* is frequently found as contaminant on surfaces within hospital environments and prolonged survival on these surfaces has been reported. For transmission, a small inoculating dose is sufficient [158]. Vegetative *C. difficile* bacilli could survive on dry surfaces (glass) for only 15 min while survival on moist surfaces has been detected for 6 h [88,89]. Studies on survival of *C. difficile* spores showed that the spores are more likely resistant toward drying, heat, and chemical agents compared to vegetative bacilli and thus were capable to survive longer on glass slides than the vegetative forms [88,159]. It has been shown by Kim et al. [160] that *C. difficile* inoculated onto floor material survived for five months [160]. Additionally, Otter et al. showed that *C. difficile* was able to persist on stainless steel for more than six weeks with no significant loss in titer [87].

Survival of *C. difficile* has been reported to range from minutes as on glasses to months as on floor materials (Table 3). A small dose is required for sufficient transmission, thus implicating a potential risk for transmission via contaminated inanimate surfaces.

##### *Listeria monocytogenes* 

The foodborne pathogen *Listeria monocytogenes* is linked to infections of the central nervous system and maternal-neonatal listeriosis [161]. Infections with listeria is associated with high morbidity and case fatality rates; thus, studies on *L. monocytogenes* virulence and human listeriosis surveillance programs have been carried out in numerous amounts. Also, survival analyses of *L. monoytogenes* on several inanimate surfaces have been proceeded.

Helke and Wong examined the survival of *L. monocytogenes* on stainless steel and buna-n^®^ rubber, inoculated in PBS and milk. On stainless steel and on buna-n^®^ rubber, the time until the bacteria were undetectable were determined to be between three and more than 10 days. While it has been found that the inoculation in PBS and whey, a temperature of 25 °C and a low relative humidity (32.5%) had negative effects on persistence, the inoculation in milk, a temperature of 6 °C and a high relative humidity (75.5%) increased the survival time [98]. Survival studies on stainless steel reported by Hansen and Vogel revealed comparable results: *L. monocytogenes* was able to survive for more than 23 days. Additionally, the survival of biofilm associated *L. monotcytogenes* on stainless steel has been evaluated. Extending the investigation period to 49 days, it has been shown that the life form biofilm had beneficial effect on survival. While a biofilm—especially an osmo-adapted one—had a positive effect on survival, the concentration of tryptic soy broth with 1% glucose (TSB-glu) had ambiguous effects [99].

Vogel et al. showed that *L. monocytogenes* was able to survive on stainless steel for more than 91 days. TSB-glu enhanced the survivability more than physiological peptone saline (PPS) and salmon juice. Furthermore, relative humidity had ambiguous effects on persistence [100]. Nese et al. [102] could show that *L. monocytogenes* was able to survive for at least 180 min on wood and plastic. On plastic, the survival was significantly better than on wood [102].

Huang et al. [162] examined the effect of sigB on desiccation survival of *L. monocytogenes*. *Listeria* persistence was not affected by ΔsigB except in minimal medium. In all tested conditions, the bacterium was able to persist for more than 21 dys [162].

*L. monocytogenes* has been shown to persist on inanimate surfaces in a material dependent manner. Longest survival has been shown in studies on stainless steel with persistence up to 91 days (Table 3).

##### Other Gram-Positive Bacteria

*Micrococcus* species are usually recognized as contaminants of the human skin. However, they have been detected as the cause of various infectious diseases, among others bacteraemia and endocarditis. The number of publications reporting on survival of micrococcacea on inanimate surfaces is low. However, there are two studies dealing with *Micrococcus luteus* survival [163]. It has been demonstrated that *M. luteus* survives for at least one day on cotton lint, [96]. Zeidler and Müller could show, that *M. luteus* is inactivated after 150 days on polycarbonate membrane filters [86].

One study did report on survival properties on inanimate surfaces of pathogenic Gram-positive bacteria *Corynebacterium diphtheriae*. Even though infections with *C. diphtheriae* mainly occur in underdeveloped countries, its impact on human health is imminent. There have been only low numbers of reports on *C. diphtheriae* survival. According to Crosbie and Wrigth, *C. diphtheriae* can survive in floor dust for up to 102 days [90].

*Bacillus subtilis* is a sporulating bacteria that is ubiquitous in the environment and its pathogenic potential is widely described as low or absent [164]. However, in the literature, a few cases of human infections caused by *B. subtilis* are reported, among them a nosocomial infection outbreak in Japan [165,166,167,168]. Survival studies of *B. subtilis* on inanimate surfaces are thus also more rarely published. Zeidler et al. showed a long-term survival of *B. subtilis* on polycarbonate membrane filters with a 1–2-log titer reduction after 200 days [86].

#### 3.4.2. Gram-Negative Bacteria

Infections caused by gram-negative bacteria are frequently occurring. Among the most relevant gram-negative human pathogens are *Klebsiella pneumoniae*, *Salmonella* ssp, and *Escherichia coli*.

##### *Escherichia coli* 

*Escherichia coli* is a well-studied model organism for gram-negative bacteria. The Shiga-toxin and Shiga toxin-like producing *E. coli* is an important foodborne pathogen, causing the hemolytic-uremic syndrome especially in children. Additionally, the gut bacteria *E. coli* is found to cause bloodstream infections associated with hospitalization [169]. Survival analyses of *E. coli* on several inanimate surface have been published in numerous amounts.

Among others, Scott et al. examined the survival of *E. coli* on laminate and j-cloth^®^. *E. coli* could survive on laminate for up to 24 h, although the titer was diminished significantly after 4 h [104]. A comparison of wild-type strains with laboratory strains revealed improved survivability of wild-type strains and a beneficial effect of soil conditions on the survival. On j-cloth^®^ and under soiled conditions, wild-type strains can persist for more than 48 h, while laboratory strains survived for 4 h [104]. Clean conditions caused an increased survival to 24 h of *E. coli* laboratory strains on cloth. Moreover, the contact with contaminated surfaces and cloths has been found to represent a potential hazard if in contact with food sources [104]. Also, others, among them Neely, tested the survival of several gram-negative bacteria on cloths. *E. coli* was inactivated on cotton, terry, a cotton-polyester blend and spandex at the latest after 2 days. In contrast to that, it survived on polyester for up to 9 days and up to 25 days and 36 days on polyethylene and polyurethane, respectively [129]. Hirai showed that *E. coli* was inactivated on cotton lint after 7 days [96].

According to Abrishami et al, *E. coli* was able to survive on wood for at least 2 h [133]. At that time point, the calculated log reduction did not exceed 1.7 logs, so it can be assumed that the viability on wood is far longer than 2 h. The survivability on used wood was increased in comparison to fresh wood [133]. Comparable results have been published by Ak et al., who detected a ~4-log reduction after 2 h on wood [102]. Montibus et al. detected survivability of *E. coli* on wood for at least 7 days [138]. On plastic, *E. coli* was still viable after 24 h without showing any reduction in titer [133]. Another study revealed persistent *E. coli* on plastic for more than 16 days at 4°C [134]. Hokunan et al. examined the effect of temperature and relative humidity on the persistence of three *E. coli*-strains (O26, O111, O157:H7) on plastic. The authors could show that an increasing temperature led to a more rapid bacterial decay. The strains seemed to be relatively resistant to most RHs—except very high RH (93%). However, very low RHs were not studied. The longest survival was detected at 5 °C and RH < 93%, where the bacteria persisted for more than 300 days without a significant loss in titer. At 25 °C, all strains were inactivated after 100 days [135]. On a polymer and at 37 °C, *E. coli* persisted for 24 h as shown by Kampf et al. [105]. When the polymer contained silver, *E. coli* was still detectable after 7 days in small amounts [105]. Maule reported that *E. coli* was not inactivated after 60 days on stainless steel at 4 °C. At the same conditions *E. coli* was not detectable after 10 h on copper [134].

Wilks et al. studied the effect of copper on survivability of *E. coli*. The authors found a quick bacterial inactivation on copper alloys at 20 °C: no viable strains were detectable after 90 min on copper surfaces. In contrast to that, viable bacteria were still detectable after 28 days on stainless steel, showing a 5-log reduction. Copper nickel and copper zinc alloys take a middle ground between copper and stainless steel. A lower temperature (4 °C) decelerated the bacterial decay [136].

Williams et al. compared the persistence of *E. coli* O157 on several metal and wood surfaces [137]. Generally, the authors found that a low temperature (5° C) and a moist environment as well as a wood surface to increase survival. A higher temperature (20 °C), a desiccated environment, and a metal surface accelerated the bacterial decay. In a moist environment, *E. coli* survived in all conditions for more than 28 days—except at 20 °C on metal surfaces where it was inactivated after 7 days. The shortest persistence was documented at 20 °C on metal and in a desiccated environment, where the strains were already inactivated after 3 days.

In contrast to *S. aureus*, where all bacterial strains survived on ceramic floor, synthetic fiber, cotton and mattress for more than eight weeks, *E. coli* was not so stable on all materials, Esteves et al. showed. While *E. coli* was also able to persist on floor and mattress for more than 8 weeks, it was inactivated after four weeks and eight weeks on synthetic fiber and cotton, respectively [97]. In comparison to some enterococci, *E. coli* only persisted for 14 days on glass slides, Bale et al. showed [93]. According to Jawad et al, *E. coli* was only detectable for 1 day on glass when suspended in distilled water, slightly increased to 3 days in BSA [111]. On polycarbonate membrane filters, *E. coli* DH5α were still detectable after 6 h, although showing a 6-log reduction at that time [86].

The current available literature reporting on *E. coli* survival on different inanimate surfaces is numerous. It is assumed that *E. coli* persistence is dependent on several factors. Next to strain specific characteristics and material nature, also environmental conditions as temperature and humidity have been shown to exhibit a huge impact on survivability duration.

##### *Klebsiella pneumoniae* 

*Klebsiella* spp. are frequently found to cause nosocomial infections, especially in immunocompromised patients suffering from severe underlying diseases. Most of the Klebsiella infections associated with hospitalization are caused by *Klebsiella pneumoniae* [170]. A few studies dealing with survival properties of *K. pneumonia* on inanimate surfaces have been published. While Hirai stated that *K. pneumoniae* was inactivated after 1h on cotton lint [96], Neely showed that the bacteria were able to survive for at least 4 days on fabrics—including cotton—and up to 27 days and 32 days on polyethylene and polyurethane, respectively [129]. The results of Esteves et al. presented a bacterial survival of *K. pneumoniae* on cotton and synthetic fiber for four weeks. The bacteria also survived on ceramic floor and mattress for two weeks and three weeks, respectively [97]. Furthermore, Otter et al. showed that *K. pneumoniae* was able to survive on stainless steel for 3–6 weeks. The strains persisting for > 6 weeks showed a 4–5-log reduction in titer [87].

Different survival durations have been detected in the above-named studies for *K. pneumoniae,* particularly when persistence on cotton has been analyzed, thus implicating that survival of *K. pneumoniae* is dependent on various conditions and is probably also strain specific.

##### *Salmonella enterica* 

*Salmonella* spp. causes regularly occurring gastrointestinal infections after uptake of contaminated water or foods, being particular clinical relevant as one of the most common source of food-borne illness worldwide [171]. Especially in elderly or immunosuppressed patients, the infections are severe. *Sallmonella enterica* serovar Typhimurium is one of the most important serotypes of *Salmonella* spp. and is linked to a transmission from animal to the human host.

Survivability of *S.* Typhimurium on inanimate surfaces was first reported by Robertson in 1972. When kept in ward dust, strains were detectable for at least four years and two months [143]. In contrast, the surface of cotton resulted in much shorter lifespan of the bacteria with 5 h of survival [96].

As transmission of *Salmonella* is linked to food sources, studies on survival capabilities of *Salmonella* in different groceries have been published. Among them a study from Helke and Wong, stating that *S*. Typhimurium survival is positively impacted by low temperatures (here 6 °C), high RH (75.5%) and suspension in milk. In contrast, a temperature of 25 °C, low RH (32.5%), and suspension in PBS or whey led to a faster inactivation. Additionally, also the surfaces impacted survival duration. Generally, survival has been detected to be much longer on stainless steel than on buna-n^®^ rubber. The fastest inactivation was found on buna-n^®^ rubber at 25 °C and 32.5% RH, where there were no bacteria detectable after 1 day. In milk, most bacteria persisted for more than 10 days [98]. The effect of lowering temperature has also been found by Hokunan et al., who showed that *S.* Typhimurium persisted on plastic for more than 300 days, when kept at 5 °C [135]. At 15 and 25 °C, the strains were viable for up to 170 days and 90 days, respectively. Also, a humidity-dependent survival has been detected. However, in contrast to Helke and Wong, Hokunan et al. found a high RH of 93% leading to a faster bacterial decay as shown for Salmonella on plastics [135]. Influence of environmental conditions such as medium have further been eva luated by Lesne et al. who examined the survival of *S.* Typhimurium on polycarbonate membranes [147]. The bacteria were detectable for 7–8 weeks—depending on the medium used before [147]. Also, the growth phase of bacteria inoculated on the surface has been shown to have an impact on survival duration. While early and mid-log bacteria were detectable for at least eight weeks, stationary and plate-derived bacteria were even detectable after 12 weeks at 4 °C on polystyrene- the plate-derived bacteria showed almost no loss in titer at that time point [145]. Ramachandran et al. compared the *S.* Typhimurium-strains ST313 and ST19. While both strains survived for one month at RT on plastic and polycarbonate membranes, the titer of the biofilm-producing strain ST19 decreased significantly less than that of the poor-biofilm producing strain ST313 [146]. On stainless steel, long term persistence has been proven in a study including four strains of *S.* Typhimurium. Each of the strains were able to survive for more than 30 days [148]. A study of Finn et al. demonstrated consistent results with survival on stainless steel for a minimum of six weeks under desiccation stresses with a relevant role of the osmoprotectant transporter gene *proP* [144].

Next to *S.* Typhimurium, also studies on survival of *Salmonella enterica* serovar Abony and serovar Enteritidis have been published. Among others, laboratory strains of *S.* Abony were inoculated on laminate and j-cloth^®^ under simulated clean and soiled conditions by Scott et al. on j-cloth, the bacteria were able to survive for at least 48 h, regardless of the condition. In contrast to the cloth, *S.* Abony persisted on laminate for up to 24 h in a soiled condition, but was inactivated after 4 h in a clean condition [104].

The survival of *Salmonella enterica* serovar Enteritidis on almonds was studied by Uesugi et al. [142]. The authors could show that *S. enteritidis* could survive for at least 171 days. At 4 °C, the titer reduction was lower than at 23 or 35 °C, confirming the temperature-dependent survival capabilities of *Salmonella* [142]. On cotton lint, *S.* Enteritidis persisted for up to 1 h, according to Hirai [96]. Margas et al. showed that *S.* Enteritidis persisted for more than 30 days on stainless steel [148].

Other *Salmonella* spp. have also been tested on their survival properties on inanimate surfaces. Among others, survival of not further defined wild-type *Salmonella* was analyzed by Scott et al. [36]. They detected viable *Salmonella* for more than 24 h on laminate and for more than 48 h on j-cloth^®^ [36]. According to Margas et al., a total of 15 *Salmonella* isolates –including strains of the *Salmonella enterica* serovars Typhimurium and Enteritidis—survived for more than 30 days on stainless steel [148]. Together with three *E.* coli-strains and *S.* Typhimurium, Hokunan et al. also studied the survival of three other *Salmonella enterica* serotypes, namely *S.* Chester, *S.* Oranienburg, and *S.* Stanley at three temperatures (5, 15, 25 °C) and five different RHs. As found with *E. coli* and *S.* Typhimurium, the three strains persisted generally longer at lower temperatures and the loss of viability was faster at a high relative humidity (93%). Interestingly, *S.* Chester and *S.* Oranienburg survived longer at 25 °C than at 15 °C. The longest survival of all three species were detected at 5 °C, where the bacteria were not inactivated after 300 days and showed a maximum reduction of 4 logs [135]. Other serotypes, *S.* Tennessee and *S.* Eimsbuettel were detectable after 72 h on plastic at 22 °C and 40% RH according to a study by Abdelhamid et al [149]. The titer reduction was 1-log after 72 h [149].

A high number of survival studies on *Salmonella* have been published so far. The evidence that certain environmental conditions are impacting survival properties of *Salmonella* is given in various publications. Temperature, RH and also surrounding medium are influencing survival capability. Particularly presence of milk products is beneficially affecting *Salmonella* survivability, thus implicating one reason for the frequently occurrence of the foodborne illness of *Salmonella* infections. The survival of *Salmonella enterica* has been detected to be serotype related.

##### *Pseudomonas* spp.

*Pseudomonas aeruginosa*, one prominent example of frequently occurring gram-negative bacteria, is associated with hospital acquired infections due to its capability to survive under challenging environmental conditions [172]. Neely presented that *P. aeruginosa* was viable for up to 3 days on cloths and up to 10 days on plastics [129]. Esteves et al. showed a much longer survival of *P. aeruginosa* on ceramic floor, synthetic fiber, cotton, and mattress with survival for more than eight weeks [97].

Examination of *P. aeruginosa* survival using several strains, revealed a persistence on plastic ranging between 9 h and more than 48 h in a strain-dependent manner as demonstrated in a study of Panagea et al. [141]. According to Webster et al., *P. aeruginosa* was viable for 4 days on Formica, 5 days on stainless steel and 1 day on a drip stand [110]. On a polymer and at 37 °C, *P. aeruginosa* has been shown to persist for more than 7 days independent of the presence or absence of silver [105]. It has been shown by Scott and Bloomfield that *P. aeruginosa* persisted for at least 24 h on laminate and 48 h on j-cloth^®^, respectively. A suspension in tryptone soy broth did not affect the bacterial survival or titer reduction [104]. Hirai showed that *P. aeruginosa* was inactivated after 2 h on cotton lint while on glass, viable *P. aeruginosa* could not be detected after 7 h [96].

Further comparing survival of *P. aeruginosa* with *Pseudomonas cepacia,* the latter has been detected to be less persistent on glass (5 h) and cotton lint (2 h) [96].

Additionally, to *P. aeruginosa* and *P. cepacia*, one study did also report *on Pseudomonas putida* survival on glass. *P. putida* was inactivated on glass after 2 days [93].

*Pseudomonas* spp. have been found to survive on several inanimate surfaces in a material-, species- and strain-dependent manner. The detected survival durations are ranging between 2 h on cotton and 8 weeks on ceramic floor.

##### *Acinetobacter* spp.

A broad range of different diseases have been linked to infections with bacteria belonging to the genus of *Acinetobacter*. Mainly associated with nosocomial infections and most frequently human infecting *Acinetobacter* species is *Acinetobacter baumannii*. Numerous outbreaks with *A. baumannii* in intensive care units have been reported [173]. Jawad et al. examined the persistence of several *A. baumannii*-strains on glass and showed a range of survival between 2 and 30 days in distilled water [111]. Furthermore, the mean survival time was much longer with increasing RH from 28–34% than at 9–11% RH, with 20 days and 3 days, respectively [118]. To evaluate if outbreak strains were more resistant against desiccation than sporadic strains, Jawad et al. tested the survival time of 22 outbreak and 17 sporadic strains. With a mean survival time of 27.29 days and 26.55 days for sporadic and outbreak strains, no significant difference was detected [119]. Other studies on glass revealed *A. baumannii* persistence between 7 and more than 100 days in a strain dependent manner [120,121,122]. In this context, biofilm forming *A. baumannii* strains have been detected to survive significantly longer (>35 days) on glass than non-biofilm forming strains (15 days) [121]. The strain-dependent survival could also be demonstrated for other surfaces as plastics. Farrow et al. found a difference between certain strains which was substantial: While strain ATCC 19606 only had a mean survival of 3 days, strain ATCC 17961 was still detectable after 90 days [123]. Greene et al. studied the survival of *A. baumannii* strains on plastic and compared its ability to form a biofilm and the presence or absence of a multi-drug resistance (MDR). The authors found that the bacteria were inactivated at earliest after 56 days. Furthermore, a MDR and the ability to form biofilms led to an increased survival [124]. These results are comparable to those published for survival on glass surfaces.

On Formica, stainless steel and a drip stand, *A. baumannii* survived for 11, 12, and 6 days, respectively, Webster et al. showed [110]. According to Zeidler and Müller [86], *A. baumannii* were able to persist on polycarbonate membrane filters for at least 20 days to more than 28 days, depending on the used media and washing substances [86].

Tipton et al. examined the relevance of the capsule for desiccation tolerance [125]. They found that in a 12 days investigation period, the loss of a capsule led to a significantly faster bacterial decay than in the control [125]. To compare the capsule types of *A. baumanni*—virulent opaque (VIR-O) and avirulent translucent (AV-T) cells, Chin et al. inoculated plastic with both capsule types [126]. While both phenotypes persisted for more than 8 days, VIR-O was more stable than AV-T cells at dry surfaces. Furthermore AV-T cells additionally converted into VIR-O cells when exposed to desiccation conditions [126].

Next to *A. baumannii* which has former been designated as *Acinetobacter calcoaceticus* var. anitratus, other *Acinetobacter* species are, even though occurring more rarely, also linked to nosocomial infections. Musa et al., investigated the survival of *A. calcoaceticus* var. *Iwolfii* and compared it with *A. baumannii* survival capabilities. While var. *Iwolfii* persisted for 24 h on Formica, the survival of *A. baumannii* was detected to be between 48 h and 72 h [127]. This study stands in contrast to an investigation published by Getchell White et al. who found a median survival on Formica of var. *lwoffii* of 10.2 days and of *A. baumannii* of 8.2 days [128]. Hirai compared also the survival on both species but investigated another material, being glass and cotton lint. *A. baumannii* did survive on glass for more than 7 h and on cotton lint more than 25 days whereas var. Iwolfii was inactivated on cotton lint already after an investigation period of 7 h [96]. On glass and polypropylene, *A. calcoaceticus* was able to survive for at least 14 days [93]. Jawad et al. found that *A. calcoaceticus* survived for 22–23 days on glass, when kept in distilled water. The mean survival was subsequently longer when kept in bovine serum albumin, indicating a beneficial effect of nutrient supply [111]. Jawad et al. further found that on glass *A. calcoaceticus* var. *lwoffii* only persisted for 18 h in average at 10% RH and one to six days at 31% RH [118], confirming previously described beneficial effect of increased RH on survival capabilitites.

In contrast to var. *lwoffii*, *A. radioresistens* had a mean survival time on glass of 157 days at 31% RH and 31 days at 10% RH, as Jawad et al. showed [118].

Not further specified clinical strains of *Acinetobacter* were evaluated on Formica, stainless steel, and a drip stand by Webster et al. Survival was stated to be 6 days on stainless steel and Formica and 3 days on the drip stand [110]. Another investigation revealed a survival of *Acinetobacter* of six weeks on stainless steel, this being even longer than the survival duration described by Webster et al. [87]. Neely showed that *Acinetobacter* spp. were able to survive for up to 14 days on cloths and for more than 60 days on polyethylene and polyurethane [129].

*Acinetobacter* survival has been shown to vary widely on different materials and between species. *A. baumannii*, the most prominent *Acinetobacter* species causing infections in humans has been demonstrated to be most persistent on glass. It has also been shown that the capsulation of *A. baumannii* is a key factor in persistence capability.

##### Other Gram-Negative Bacteria

*Neisseria gonorrhoeae* is known to cause the sexually transmitted infection gonorrhea, being a global public health concern with increasing numbers of reports on antibiotic resistances [174]. Even though main transmission of *N. gonorrhoeae* takes places via sexual contacts, also studies on persistence on inanimate surfaces have been performed. Pérez et al. were able to show that *N. gonorrhoeae* could survive for at least 24 h on plastic and bed sheets. After 24 h, the remaining amount of bacteria was 9.1% and 8% on plastic and bed sheets, respectively [140].

*Francisella tularensis* is the cause of tularemia and has been reported to be isolated from birds, reptiles, fish, invertebrates, rodents, amoeba and higher mammals [175,176]. Human-to-human transmission has not been reported for tularemia, it is rather associated with contact to infected animals. One study reporting on survival of *F. tularensis* was published. Richter et al. examined the survival of two (Schu4 and LVS) *F. tularensis* strains on glass and paper at several temperatures and distinct RHs. Generally, *F. tularensis* strain Schu4 had a better survivability than the strain LVS. Both strains persist longer on glass than on paper at almost all tested conditions. A rising temperature and RH led to a decreased survival time. The longest survival was seen at 38 ± 2 °C and 41 ± 5% relative humidity: both strains were still detectable after 10 days on glass. On paper, Schu4 was inactivated after 96 h and LVS after 24 h [139].

*Shigella dysenteriae* is associated with diarrheal diseases, transmitted predominantly via unsafe drinking water. *Shigella* is a highly infectious human pathogen from which as small as ten organisms are enough to cause disease in human [177]. Islam et al. examined the survival of *S. dysenteriae* on several surfaces. The bacteria survived for 3 h on wood and 4 h on cloth. Furthermore, the persistence accounted for 2 h on glass and aluminum and 1.5 h on plastic [150].

*Proteus mirabilis*, commonly found in human feces, is known to be a causative agents of urinary tract infections [178,179]. Together with other gram-negatives, Neely examined the survival of *P. mirabilis* on several cloths as well as plastics. On cloths, the bacteria persisted between 4 h and 9 days. The survival on polyethylene was similar to those on cloths, but on polyurethane, *P. mirabilis* was able to survive for up to 26 days [129].

*Serratia* spp., especially *S. marcescens,* cause mainly nosocomial infections and outbreaks are reported [180,181]. Neely showed that *S. marcescens* persisted on cotton, terry cloth and a cotton-polyester blend for up to 3 days. In contrast to that, the survival on polyester and spandex was slightly longer with 6–7 days, and even longer on polyethylene and polyurethane with up to 10 days [129]. According to Hirai, *S. marcescens* was inactivated on cotton lint after just one h, but was still viable after 7 h on glass, although showing a 2-log reduction in titer [96]. Furthermore, Jawad et al. detected *S. marcescens* for 10 days on glass in distilled water [111].

Infections with *Bordetella pertussis* have been shown to be endemic in adolescents and adults. Symptomatic adolescent are the main source of infections in unvaccinated children [182]. Hahn and Janßen wrote in an overview article on *Bordetella* that *Bordetella pertussis* were able to survive in dust and on plastic and cloth for 3–5 days without being specific about methods or providing a source [130].

*Campylobacter jejuni* is one major source of gastroenteritis and cases of C. *jejuni* infections are reported worldwide [183]. Two studies on *C. jejuni* survival properties on inanimate surfaces have been published: *C. jejuni* were inactivated within 2 h, when dried on filter paper, Luechtefeld et al. stated [131]. Oosterom et al. showed that *C. jejuni* when dried rapidly was inactivated after 15–30 min on aluminum, stainless steel, ceramics, and Formica, whereas when dried slowly, it was detectable for 7 h [132]. An explanation was provided by the authors: The first time when the bacteria were not detectable anymore, was the time point where the inoculum was visibly dried [132].

Additionally, another human relevant pathogen is the bacterium *Stenotrophomonas maltophilia*. *S. maltophilia* is mainly associated to cause nosocomial infections in immunocompromised patients but is also found as the cause of community acquired infections [184,185]. Survival analysis showed that *S. maltophilia* persisted on several cloths for 7 days [84].

#### 3.4.3. Other Bacteria

While a lot is published on gram-positive and gram-negative bacteria persisting on several inanimate surfaces, less has been published on other bacteria such as *Mycobacterium bovis*, *Chlamyida pneumoniae* and *Chlamydia trachomatis*. The data described below are further listed in Table 5.

By now, only one study investigated the survival of *M. bovis*. Hirai [96] described that *M. bovis* is viable for at least two months on cotton lint [96].

While Falsey and Walsh showed that *C. pneumoniae* survived on tissue paper for 12 h and on Formica for even 30 h [151], Novak et al. analyzed the survival of *C. trachomatis* on plastic. *C. trachomatis* persisted for 30 min and 120 min on plastic at 56% and 100% RH, respectively [152]. Generally, the survival durations of *Chlamydia* spp. have been shown to be shorter than described for other human pathogenic bacteria. Additionally, it is to mention that the increase of RH is not affecting the persistence of *C. trachomatis*. However, more studies on Chlamydia and Mycobacterium survival are necessary to provide reliable data.

### 3.5. Fungi

Globally, several billions of people are infected every year by fungi. Even though the pathogenic potential of numerous fungal species is unknown and other species are known to cause only harmless infections, a few fungi have been reported to frequently cause fatal infections in humans [186].

Especially immunocompromised patients, such as individuals suffering from HIV infections, cancer or organ transplant patients are at higher risk to develop more severe forms of fungal infection. Particularly, immunocompromised patients are often hospitalized; thus, the transmission of fungi from hospital-associated surfaces is from interest. Survival studies involving yeast and filamentous fungi on inanimate surfaces are summarized below. The findings are further listed in Table 6.

#### 3.5.1. Filamentous fungi

The most often clinically associated filamentous fungi belong to the genus *Aspergillus*. This genus is characterized by high diversity and contains over 330 species that occur worldwide in various habitats [198]. Aspergillosis is the term of infectious diseases caused by Aspergillus—among them are noninvasive infections of the respiratory tract, ears, or eyes. Additionally, also invasive infections with *Aspergillus* are reported, mainly occurring after surgery or upon immunosuppression. These are considered to cause more likely lethal forms of aspergillosis [199] The most clinically relevant *Aspergillus* species is *Aspergillus fumigatus*.

Literature research revealed a few studies dealing with *A. fumigatus* survival on surfaces. While the study group around Weave et al. demonstrated a persistence on aluminum and copper for 120 h [188], others studied the survivability on cloths and plastic. Koca et al. revealed the capability of *A. fumigatus* to survive on cotton, cotton-polyester and wool for more than 30 days and on silk for 27 days [92], which could be proven in a study of Neely and Orloff with survival of 1 to at least 30 days on both, cloths and plastics [187]. Similar survivability on cloths and plastics have been described for *Aspergillus niger* and *Aspergillus flavus* [187]. In contrast to *A. fumigatus*, *A. flavus* showed only 96 h persistence on copper and *A. niger* a survivability for more than 567 h, while aluminum as the evaluating surface revealed for each of the *Aspergillus* species a persistence of more than 120 h [188]. *A. flavus* additionally has been analyzed on its persistence on a cardboard in a study from Siroli et al., where they showed a longer survivability on cardboard in humid conditions (more than 48 h) than in dry conditions (less than 24 h) [103]. Also flooring materials have been shown to be an inanimate surface suitable for long survivability of *Aspergillus*. A study on *A. niger* survivability on flooring materials including wood, porcelain tile and vinyl demonstrate that *A. niger* is capable to survive for at least 28 days on these materials. In contrast, *A. niger* spores were undetected on both, residential broadloom- and rubber-backed carpets after 2 days [189].

*Aspergillus* spp. has been shown to persist for periods up to 30 days on different inanimate surfaces in a surface- and species-dependent manner. Additionally, it could have been demonstrated that the humidity is influencing the persistence capability of *Aspergillus*.

Next to *Aspergillus*, also other filamentous fungi are known to cause infections in humans among them *Geotrichium candidum* and *Penicillium* spp.

In a study by Koca et al., *G. candidum* has been detected to survive for 6 days on cotton-polyester and silk, 12 days on wool and for 21 days d on cotton [92]. Other fungal species as *Penicillium crysogenum* have been detected to survive on aluminum much longer (>120 h) compared to copper (6 h) [188]. *Penicillium expansum* persisted in another examination from Monitbus et al., for more than 7 days on wood and also on apples, which is similar to the results obtained for the bacterial pathogen *E. coli* [138].

*Fusarium* is a large genus comprising several species. Most of them are plant and soil associated and harmless for humans. Other species have been connected to produce mycotoxins, potentially affecting human health. Infections by *Fusarium* are linked to an uptake of the fungi via the food chain [200]. Among the clinically relevant *Fusarium* species is *Fusarium solani*. Together with the plant pathogens *Fusarium culmonium* and *Fusarium oxysporium*, it has been reported to survive for at least 120 h on aluminum while inactivated on copper after 48 h [188]. *Fusarium verticilloides* has been shown to persist in maize residues for at least 630 days as detected in a long term study by Cotten and Munkvold [197]. Additionally, another study with not further defined *Fusarium* species has reported on persistence on cloths for 10 days, on polyurethane for 6 days and on polyethylene for more than 30 days [187]. Mucormycosis, an invasive fungal infection caused among others by species belonging to the genus of *Mucor*, is known to be often severe in immunocompromised patients [201]. In a survival analysis by Neely and Orloff [187] *Mucor* ssp. was detected to persist for 16 to 24 days on most of a broad range of tested cloths and plastics with the exception of spandex. On spandex, *Mucor* survived even longer than 30 days [187]. Neely and Orloff additionally studied survival properties of *Paecilomyces* spp., which is ascertained to be a rarely occurring human pathogen [202]. On cloths and plastics, resulting in persistence of less than 1 day on cotton, terry and cotton-polyester mix. On polyester, spandex, polyethylene, and polyurethane, it survived for 5, 11, 4, and 11 days, respectively [187].

#### 3.5.2. *Candida* spp.

*Candida* spp., belonging to the yeasts, is known to be a commensal fungus which is commonly found on the human skin, mouth or in the gastrointestinal tract. *Candida* spp. is able to elicit opportunistic infections called candidiasis, sometimes becoming invasive with the potential to cause systemic infections of blood- or tissue- candidiasis. *Candida* spp. is claimed to be the most common causative agent of fungal infections in humans, with estimated 50,000 death per year, especially occurring among hospitalized patient [203].

Persistence of *Candida* has been studied on several surfaces with most data on *Candida albicans*, which is the most described cause of candidiasis. As before already described for filamentous fungi, growth on copper resulted in less long survival than growth on aluminum [188]. Going along with these results, Quaranta et al. did link the shortened survival on copper to a fungicide effect of copper alloys when comparing survival of *C. albicans* on copper with survival on stainless steel. While *C. albicans* did persist for more than 1 h on stainless steel and for a maximum of 60 min on nordic gold, the fungus persists for only 5 min and 30 min on copper and cupronickel respectively [190]. Other studies on stainless steel revealed a persistence capability of more than 24 h to 7 days at RT [191,192] and persistence of less than 6 h at body temperature [193]. In contrast to copper, silver impregnation did not work as a fungicide against *C. albicans*, as comparison of *C. albicans* persistence on a polymer being silver impregnated or not reveal no difference and survival in both cases has been at least 7 days [105]. Survival studies on *C. albicans* and textiles showed persistence of 6 days on cotton, and cotton-polyester and 12 days on wool and silk [92]. Other even defined survival for at least 14 d on both, silk and cotton [191]. Neely and Orloff determined *C. albicans* survival on cloths to be more short ranging from 1 to 4 days and longer survival with up to 6 days on plastics [187]. The survival on plastics has further been studied by Rangel-Frausto et al., resulting in persistence for at least 24 h [194]. On glass, *C. albicans* survived 3 days [191].

One *Candida* species increasingly recognized is *Candida auris* after first described in 2009 [204]. *C. auris* is described as an emerging multi-drug resistant fungal pathogen, causing mainly nosocomial infections associated with high mortality [205]. Survivability on plastic surfaces have been published by two different research groups. Welsh et al. detected viable *C. auris* on plastics for at least 14 days [195], agreeing with a study of Short et al. [196]. An assay measuring the esterase activity as parameter for living cells revealed even survivability of more than 28 days on plastics [195]. On stainless steel, Piedrahita et al. detected *C. auris* survival for more than 7 days [192].

Other clinically relevant *Candida* species are *Candida glabrata*, *Candida krusei*, *Candida parapsilosis*, and *Candida tropicalis*. Researchers around Koca studied the survival of *C. glabrata*, *C. krusei*, *C. parapsilosis*, and *C. tropicalis* on cotton, cotton-polyester, wool and silk. While they found *C. glabrata* and *C. parapsilosis* being vital on each of the tested surfaces for at least 30 days, *C. krusei* and *C. tropicalis* survived only on wool for more than 30 days. On cotton, cotton–polyester, and silk, survival of *C. krusei* has been determined to be 3, 6, and 21 days and survival of *C. tropicalis* 3, 9, and 24 days, respectively. [92] Results regarding *C. parapsilosis* survival on cloths and plastic confirmed the study of Neely and Orloff from 2001 [187]. Neely and Orloff also demonstrated the survival of *C. krusei* being shortened on cotton compared to other materials [187]. The fungus survived for less than a day on terry, for 1 day on cotton and for 4 days on a cotton–polyester blend while survival analysis on polyester and spandex showed 8 days and 11 days survival respectively. On polyethylene, *C. krusei* survived 7 days and on polyurethane for 4 days [187]. Survival of *C. parapsilosis* on cotton, cotton–polyester, steel, and glass have been shown to be at least 7–14 days [188,192] and on plastic more than 28 days [195]. *C. tropicalis* survival has been studied by Neely and Orloff, on cloths to be 1–2 days on cotton and terry, 3–5 days on cotton-polyester blend and on polyester and spandex, the fungus persisted for 8 days and 10 days respectively. Longest persistence has been detected to be on polyethylene and polyurethane [187].

*Candida* has been detected to persist between a few minutes and a month on various surfaces. While cloths have generally been detected to cause a more shortened survival, other surfaces as plastics and steel result in an extended persistence of *Candida*.

#### 3.5.3. Other Clinically Relevant Yeasts

Infections with the opportunistic yeast *Cryptococcus neoformans* are frequently reported to occur in immunocompromised patients [206]. It has been demonstrated that *C. neoformans* is capable to survive at least 30 days on cotton, cotton–polyester, silk, and wool [92].

The more rarely as a human pathogen detected baker’s yeast *Saccharomyces cerevisiae* has been studied on its survival by Siroli et al. [103]. It has been demonstrated that *S. cerevisiae* persists for more than 48 h on cardboard fibers and plastics [103]. As above mentioned for *Candida*, also for *S. cerevisiae* the fungicidal effect of copper has been shown: After 30 s of monitoring, the fungus was inactivated on copper alloys. In contrast, survivability of *S. cerevisiae* on stainless steel has been determined to be longer than the 5 min investigation period [190].

## 4. Discussion

In this review, we summarized the current literature of the surface-survival of various pathogens. Importantly, the interspecific and intraspecific range of survival time can vary noticeably, depending on surface material, temperature, relative humidity, and initial titer. Unsurprisingly, low temperatures are associated with longer persistence for most bacteria, fungi and viruses. while extensive research has been conducted for selected species (e.g., *E. coli*, *A. baumannii*, or norovirus), the survivability of many species remains widely unexplored. One reason for the lack of specific data is that cellular model systems to study the respective pathogens are not available. Overall, DNA viruses remain longer infectious as compared to RNA viruses (Table 1 and Table 2). Nonetheless, certain RNA viruses also remain infectious for weeks on selected surfaces (i.e., Poliovirus, Hepatits E virus, *Caliciviridae*). Many clinically relevant filamentous fungi and bacteria remain infectious on inanimate surfaces and can survive for months on dry surfaces (Table 3, Table 4, Table 5 and Table 6). In vitro studies can provide a first indication to assess the risk of transmission of a particular pathogen by fomites, however the conditions presented in various experimental studies frequently do resemble real-life scenarios (e.g., large inoculums, small surface area) and therefore require careful interpretation. For example, SARS-CoV2 remains potentially infectious on different surfaces for days (Table 2). However, the risk of transmission by fomites in real-life conditions is considered unlikely to occur, if standard cleaning procedures and precautions are enforced [207]. Furthermore, although different pathogens are readily transferred between skin and surfaces, the fraction of pathogen transferred is dependent on multiple factors including virus species and surface material. In particular, the efficiency of transfer of a pathogen between fomite and skin is an important parameter to model its potential for transmission and to implement effective hygiene measures, while avoiding unnecessary measures.

## Figures and Tables

**Table 1:** Survival of DNA viruses.

Pathogen	Material (Cluster)	Survival (Range)	References
*Adenoviridae*	Aluminum	7–>60 days	[18,19]
	Plastic	>9–49 days	[18,20]
	Stainless steel	1 h–>8 weeks	[21]
	Glass	1 h–>12 weeks	[21]
	Ceramics	1 h–>60 days	[21,19]
	Paper	7–>60 days	[19]
	Latex	7–>60 days	[19]
	Vinyl asbestos	1 h–>8 weeks	[21]
*Papillomaviridae*	Plastic	>7 days	[22]
Herpes simplex virus 1	Plastic	48 h–6 days	[20,23]
	Glass	4 h–>8 weeks	[21]
Herpes simplex virus 2	Plastic	4.5 h	[24]
Cytomegalovirus	Plexiglass	1–8 h	[25]
	Cotton blanket	1–2 h	[25]
	Gloves	15–240 min	[26]
*Poxviridae*	Glass	3 days–56 days	[21,27]
	Steel	1 day–56 days	[27]
	Cinder	1 day–56 days	[27]
	Cloth	<1 day–56 days	[27]

**Table 2:** Survival of RNA viruses.

Pathogen	Material (Cluster)	Survival (Range)	References
Rotavirus	Stainless steel	2–>12 days	[28]
	Paper	<2 h–>60 days	[19,28]
	Cloth	2–10 days	[28]
	Countertop	1 h	[29]
	Glass	9–>13 days	[30]
	Aluminum	>60 days	[19]
	China	>60 days	[19]
	Latex	>60 days	[19]
Coxsackie virus	Plastic	5 weeks	[23]
	Glass	2 weeks	[21]
Echovirus	Cellulose ester membrane	48–>168 h	[31]
Poliovirus 1	Aluminum	7–30 days	[19]
	China	<5–>60 days	[19]
	Latex	<5–30 days	[19]
	Paper	3–>30 days	[19]
	Cellulose ester membrane	48–>168 h	[31]
	Steel	4 h–>3 weeks	[32,33]
	Cotton	>3 weeks	[32]
	Plastic	>3 weeks	[32]
Poliovirus 2	Glass	2–>8 weeks	[21]
Rhinovirus	Stainless steel	4–>25 h	[34]
Hepatitis A-virus	Stainless steel	2 h–>30 days	[33,35]
	Aluminium	>60 days	[19]
	China	>60 days	[19]
	Latex	>60 days	[19]
	Paper	>60 days	[19]
	Wood	1–>30 days	[35]
*Caliciviridae*	Plastics	8 h–>168 days	[36,37,38]
	Ceramics	>168 days	[36]
	Stainless steel	<1 day–>168 days	[35,36,38,39]
	Cloths	1–14 days	[40]
	Glass	<1 day–7 days	[40]
	Copper alloys	<5 min–>2 h	[37,39]
	Wood	<1 day–>30 days	[35]
Hepatitis C-virus	Plastic	7 days–>6 weeks	[41]
	Stainless steel	5–>7 days	[42]
Influenza A-virus	Stainless steel	6 h- 2 weeks	[43,44,45,46]
	Plastics	<2 h–4 days	[23,43,44]
	Cloth	< 2 h–1 week	[43,46]
	Paper	12–24 h	[43]
	Wood	<2 h	[44]
Influenza B-virus	Stainless steel	24 h	[43]
	Plastic	24 h	[43]
	Cloth	6–8 h	[43]
	Paper	8 h	[43]
Parainfluenza virus	Stainless steel	2–>8 h	[47]
	Formica	0.5–>8 h	[47]
	Cloth	<0.5–>4 h	[47]
Hendra virus	Polystyrene	5–30 min	[48]
Nipah virus	Polystyrene	5–>60 min	[48]
Respiratory syncytial virus	Formica	5–7 h	[49]
	Gloves	1–4 h	[49]
	Cloth	0.5–2 h	[49]
	Paper	0.5 h	[49]
Ebola virus	Banknote	2–4 days	[50]
	Syringe	16–>32 days	[50]
HCoV-229E	Aluminum	6 h	[51]
	Sponge	6 h	[51]
	Latex	3 h	[51]
	Plastic	24–48 h	[20]
HCoV-OC43	Aluminum	2 h	[51]
	Sponge	<1 h	[51]
	Latex	<1 h	[51]
SARS-CoV-1 (incl. TGEV and MHV)	Plastic	72 h–9 days	[20,52,53]
	Paper	24 h	[54]
	Disposable gown	2 days	[54]
	Cloth	24 h	[54]
	Cardboard	8 h	[53]
	Copper	8 h	[53]
	Stainless steel	48 h	[53]
Transmissible gastroenteritis virus (TGEV) and mouse hepatitis virus (MHV) (SARS-CoV-1 surrogates)	Plastic	4 h–28 days	[55]
MERS-CoV	Plastic	8–48 h	[56]
	Steel	8–48 h	[56]
SARS-CoV-2	Cardboard	24 h	[53]
	Copper	4 h	[53]
	Plastic	72 h–4 days	[53,57]
	Stainless steel	72 h–>8 days	[53,57,58]
	Paper	30 min–2 days	[57]
	Wood	1 day	[57]
	Glass	2 days	[57]
	Cloth	1 day	[57]
	Surgical masks	4–>7 days	[57]
Astrovirus	China	7–60 days	[59]
	Paper	7–90 days	[59]
Human immunodefiency virus	Plastic	>7 days	[60]
	Glass	5 days	[61]
Hepatitis B-virus	Silanized tubes	>7 days	[62]
	Cotton	>14 days	[63]
	Stainless steel	>14 days	[63]
Hepatitis E-virus	Stainless steel	10 weeks	unpublished

**Table 3:** Survival of gram-positive bacteria.

Pathogen	Material (Cluster)	Survival (Range)	References
*Bacillus subtilis*	Polycarbonate membrane filters	>200 days	[86]
*Clostridioides difficile*	Stainless steel	>6 weeks	[87]
	glass	15 min	[88,89]
*Corynebacterium diphtheriae*	Dust	102 days	[90]
*Enterococcus* *casseliflavus*	Cloths	15–>90 days	[91]
	Plastics	>90 days	[91]
*Enterococcus gallinarum*	Cloths	28–>90 days	[91]
	Plastics	>90 days	[91]
*Enterococcus faecium*	Cloths	22–>90 days	[91,92]
	Glass	>77 days	[93]
	Plastics	1–>16 weeks	[91,94]
	Stainless steel	>6 weeks	[87]
	Countertop	>7 days	[95]
*Enterococcus faecalis*	Cloths	1–>90 days	[91,96,97]
	Glass	>77 days	[93]
	Plastics	1–>16 weeks	[91,94]
	Other surfaces	5 days–3 weeks	[97,95]
*Listeria monocytogenes*	Stainless steel	1–>91 days	[98,99,100,101]
	Plastic	>180 min–>10 days	[98,102,103]
	Wood	>180 min	[102]
	Cardboard	>48 h	[103]
*Micrococcus luteus*	Cotton lint	>1 day	[96]
	Polycarbonate membrane filters	120 days	[86]
*Staphylococcus aureus*	Cloths	1–>70 days	[91,92,96,97,104]
	Plastics	21 days–>3 years	[91,105,106,107,108]
	Stainless steel	6 h–> 6 weeks	[87,109,110]
	Copper alloys	30 min–>6 h	[109]
	Glass	15–25 days	[111]
	Flooring materials	>4 h–8 weeks	[104,97]
*Staphylococcus epidermidis*	Cloths	6–28 days	[96,91]
	Glass	>7 h	[96]
	Plastics	41–>90 days	[91]
*Streptococcus equi*	Wood	<1 to 13 d	[112,113]
	Metal	<1 to 3 d	[112]
	Rubber	<1 to 3 d	[112]
	Clothing	<1 to 13 d	[113]
*Streptococcus pneumoniae*	Plastic	< 3 d–1 month	[114]
*Streptococcus pyogenes*	Tomatoes	>2–>24 h	[115]
	Plastic	2–> 4 months	[114,115,116]
	Ceramic	>2–>24 h	[115]
	Stainless steel	>2–>24 h	[115]
	Glass	2–>88 h	[116]
	Metal	2–>88 h	[116]
	Latex	2–>88 h	[116]
	Wood	2–>88 h	[116]
	Reeds	24 h	[117]
	Clarinets (simulated play)	24 h	[117]
*Streptococcus salivarius*	Glass	2–>88 h	[116]
	Metal	2–>88 h	[116]
	Plastic	2–>88 h	[116]
	Latex	2–>88 h	[116]
	Wood	2–>88 h	[116]

**Table 4:** Survival of gram-negative bacteria.

Pathogen	Material (Cluster)	Survival (Range)	References
*Acinetobacter baumannii*	Glass	2–>100 days	[111,118,119,120,121,122]
	Cloths	7–19 days	[92]
	Plastics	3–>90 days	[110,123,124,125,126,127]
	Stainless steel	12 days	[110]
	Polycarbonate membrane filters	>20 days	[86]
*Acinetobacter calcoaceticus*	Plastics	24 h–>14 days	[93,127,128]
	Glass	>7 h–23 days	[93,96,111,118]
	Cotton	4–>25 days	[96]
*Acinetobacter radioresistens*	Glass	31–157 days	[118]
*Acinetobacter* spp.	Plastics	6–>60 days	[110,129]
	Cloths	1–14 days	[129]
	Stainless steel	6 days–>6 weeks	[110,87]
*Bordetella pertussis*	Dust	3–5 days	[130]
	Plastic	3–5 days	[130]
	Cloth	3–5 days	[130]
*Campylobacter jejuni*	Paper	< 2 h	[131]
	Aluminum	15 min–7 h	[132]
	Stainless steel	30 min–7 h	[132]
	Formica	30 min–7 h	[132]
	Ceramics	15 min–7 h	[132]
*Escherichia coli*	Cloths	4 h–>8 weeks	[92,96,97,104,129]
	Plastics	24 h–>300 days	[102,103,105,128,129,133,134,135]
	Steel	14–>60 days	[134,136,137]
	Copper alloys	60–>360 min	[136]
	Glass	1–≥14 days	[111,93]
	Flooring materials	1hours–>8 weeks	[104,97]
	Polycarbonate membrane filters	>6 days	[86]
	Wood	>2 h–>28 days	[102,137,138]
*Francisella tularensis*	Glass	2–>240 h	[139]
	Paper	1– 96 h	[139]
*Klebsiella pneumoniae*	Cloths	<1 h–4 weeks	[96,97,129]
	Plastics	9–32 days	[129]
	Stainless steel	3–6 weeks	[87]
	Ceramics/Flooring material	2 weeks	[97]
*Neisseria gonorrhoeae*	Plastics	>24 h	[140]
	Cotton-Polyester	>24 h	[140]
*Proteus mirabilis*	Cloths	4 h–9 days	[129]
	Plastics	<1–26 days	[129,128]
*Pseudomonas aeruginosa*	Cloths	1 h- >8 weeks	[92,96,97,104,129]
	Glass	5 h	[96]
	Plastics	9 h–10 days	[105,110,128,129,141]
	Stainless steel	5 days	[110]
	Flooring materials	1 h–>8 weeks	[104,97]
*Pseudomonas cepacia*	Glass	5 h	[96]
	Cotton	2 h	[96]
*Pseudomonas putida*	Glass	<2 days	[93]
*Salmonella enterica* serovar Abony	Laminate	1–>24 h	[104]
	Cloth	>48 h	[104]
*Salmonella enterica serovar Enteritidis*	Almonds	>171 days	[142]
	Cotton	1 h	[96]
	Plastic	24 h	[103]
	Cardboard	1 h	[103]
*Salmonella enterica serovar Typhimurium*	Cotton	5 h	[96]
	Dust	>4 years and 2 months	[143]
	Stainless steel	<1 day–>6 weeks	[98,144]
	Plastics	<1 day–>12 weeks	[98,145,146]
	Polycarbonate membrane	>1 month–8 weeks	[146,147]
*Salmonella* spp.	Laminate	>24 h	[104]
	Cloth	24–>48 h	[104]
	Stainless steel	>30 days	[148]
	Plastic	>72 h–>300 days	[135,149]
*Serratia marcensens*	Cloths	<1 h–7 days	[96,129]
	Plastics	1–10 days	[129,128]
	Glass	>7 h–11 days	[96,111]
*Shigella dysenteriae*	Cloth	4 h	[150]
	Wood	3 h	[150]
	Plastic	1.5 h	[150]
	Aluminum	2 h	[150]
	Glass	2 h	[150]
*Stenotrophomonas maltophilia*	Cloths	7 days	[92]

**Table 5:** Survival of other bacteria.

Species	Material (Cluster)	Survival (Range)	References
*Mycobacterium bovis*	Cotton	>2 months	[96]
*Chlamydia pneumoniae*	Formica	30 h	[96,151]
	Paper	12 h	[151]
*Chlamydia trachomatis*	Plastic	30–120 min	[152]

**Table 6:** Survival of fungi on inanimate surfaces. Survival duration is given in ranges.

Pathogen	Material (Cluster)	Survival (Range)	References
*Aspergillus flavus*	Cloths	2–>30 days	[187]
	Plastics	8–>30 days	[187]
	Aluminum	≥120 h	[188]
	Copper	96 h	[188]
	Cardboard	24–>48 h	[103]
*Aspergillus fumigatus*	Cloths	1–>30 days	[92,187]
	Plastics	5–>30 days	[187]
	Aluminum	≥120 h	[188]
	Copper	120 h	[188]
*Aspergillus niger*	Cloths	1–>30 days	[187]
	Plastics	2–>30 days	[187]
	Flooring materials	>28 days	[189]
	Aluminum	>576 h	[188]
	Copper	>576 h	[188]
*Geotrichum candidum*	Cloths	6–21 days	[92]
*Penicillium crysogenum*	Aluminum	≥120 h	[188]
	Copper	6 h	[188]
*Penicillium expansum*	Wood	>7 days	[138]
	Apple	>7 days	[138]
*Candida albicans*	Stainless steel	60 min–>7 days	[105,190,191,192,193,194,195]
	Copper alloys	5 min–6 h	[188,190]
	Aluminum	≥120 h	[188]
	Cloths	1–>14 days	[92,187,191]
	Plastics	4–>7 days	[105,187,194]
	Glass	3 days	[191]
*Candida auris*	Plastics	>14 days	[195,196,197]
	Steel	>7 days	[192]
*Candida glabrata*	Cloths	>30 days	[92]
	Steel	>7 days	[192]
*Candida krusei*	Cloths	<1–>30 days	[92,187]
	Plastics	3–7 days	[187]
*Candida parapsilosis*	Cloths	2–>30 days	[92,187,191]
	Stainless steel	>7 days	[191,192]
	Glass	>14 days	[191]
	Plastics	>28 days	[187,195]
*Candida tropicalis*	Cloths	1–> 30 days	[92,187]
	Plastics	6–18 days	[187]
*Cryptococcus neoformans*	Cloths	>30 days	[92]
*Saccharomyces cerevisiae*	Cardboard fibres	>48 h	[103]
	Plastic	>48 h	[103]
	Copper	<0.5 min	[190]
	Stainless steel	>5 min	[190]
*Fusarium* spp.	Aluminum	≥120 h	[188]
	Copper	48 h	[188]
	Cloths	4–10 days	[187]
	Plastics	6–>30 days	[187]
	Maize stalk residue	>630 days	[197]
*Mucor* spp.	Cloths	16–>30 days	[187]
	Plastics	20–21 days	[187]
*Paecilomyces* spp.	Cloths	<1–11 days	[187]
	Plastics	4–11 days	[187]
